# Dose Descriptors and Assessment of Risk of Exposure-Induced Death in Patients Undergoing COVID-19 Related Chest Computed Tomography

**DOI:** 10.3390/diagnostics12082012

**Published:** 2022-08-19

**Authors:** Lejla M. Čiva, Adnan Beganović, Mustafa Busuladžić, Merim Jusufbegović, Ta’a Awad-Dedić, Sandra Vegar-Zubović

**Affiliations:** 1 Sarajevo Medical School, University Sarajevo School of Science and Technology, 71210 Ilidža, Bosnia and Herzegovina; 2 Radiation Protection and Medical Physics Department, Sarajevo University Clinical Center, 71000 Sarajevo, Bosnia and Herzegovina; 3 Faculty of Science, University of Sarajevo, 71000 Sarajevo, Bosnia and Herzegovina; 4 Faculty of Medicine, University of Sarajevo, 71000 Sarajevo, Bosnia and Herzegovina; 5 Radiology Clinic, Sarajevo University Clinical Center, 71000 Sarajevo, Bosnia and Herzegovina; 6 Healthcare Center of Sarajevo Canton, 71000 Sarajevo, Bosnia and Herzegovina

**Keywords:** computed tomography, radiation, dose, COVID-19, radiology, CTDI, DLP, REID, imaging

## Abstract

For more than two years, coronavirus disease 19 (COVID-19) has represented a threat to global health and lifestyles. Computed tomography (CT) imaging provides useful information in patients with COVID-19 pneumonia. However, this diagnostic modality is based on exposure to ionizing radiation, which is associated with an increased risk of radiation-induced cancer. In this study, we evaluated the common dose descriptors, CTDI_vol_ and DLP, for 1180 adult patients. This data was used to estimate the effective dose, and risk of exposure-induced death (REID). Awareness of the extensive use of CT as a diagnostic tool in the management of COVID-19 during the pandemic is vital for the evaluation of radiation exposure parameters, dose reduction methods development and radiation protection.

## 1. Introduction

For more than two years, coronavirus disease 19 (COVID-19), caused by coronavirus SARS-CoV-2 (Severe Acute Respiratory Syndrome coronavirus 2), has represented a threat to global health and lifestyles. It was initially epi-centered in Wuhan, Hubei Province, China, in December of 2019 [1,2,3]. The World Health Organization (WHO) proclaimed a pandemic on 11 March 2020. Since then, it has affected hundreds of millions of people worldwide as the virus spread to over 200 countries [4,5].

The first case of COVID-19 in Bosnia and Herzegovina was confirmed on 5 March 2020, followed by the declaration of the state of emergency made by the Council of Ministers and both entity governments (federal government and government of the Republic of Srpska) [6]. Pandemic outbreak peaks were noted in October–November of 2020, March–April of 2021 and January–February of 2022. COVID-19 should by no means be ignored as China is nowadays facing its biggest outbreak since 2020 [7]. Effective risk management strategies are necessary to prevent recurring outbreaks in zero community COVID-19 cases [8].

This type of virus is highly contagious which enables its rapid transmission in communities. Patients report a wide range of symptoms (fever, cough, loss of smell and taste, fatigue, dispnea, etc.) depending on the severity of illness. The preferred diagnostic tool for diagnosing COVID-19 is reverse-transcription polymerase chain reaction (RT-PCR) assay.

The role of radiologic imaging in the diagnosis and evaluation of COVID-19 has been extensively reported since 2019 [9,10,11]. The initial diagnostic procedure for suspected COVID-19 pneumonia is chest X-ray, whose findings may be inconclusive due to sensitivity and specificity [12].

Patients who suffer severe symptoms are commonly referred to CT imaging, a diagnostic modality associated with high radiation doses. However, computed tomography (CT) imaging provides usable information in patients with COVID-19 pneumonia. Since COVID-19 is not the only viral infection causing pneumonia, the CT findings are non-specific, overlapping with other infections (influenza, H1N1, SARS, MERS) [9]. Although its clinical significance has been proved, CT accounts for approximately half of the collective medical radiation dose [13]. Different imaging techniques should be used according to clinical indications. Various literature suggests that effects of COVID-19 can be evaluated with a low-dose and non-contrast protocol [9]. In accordance with the recommendations of the Fleischner Society, chest imaging should not be indicated for patients with mild symptoms of COVID-19 disease. However, it is justified in the case of worsening respiratory status or for patients with moderate to severe clinical picture with a high possibility of being infected [9,14].

The radiation burden is increased if a patient undergoes repeated CT examinations, which increases the risk of radiation-induced cancer [15]. Dose descriptors commonly used in CT dose management are air kerma length product (*P*_KL,CT_ or KLP, also abbreviated as DLP) and pitch-corrected volume CT air kerma index ($C_{\mathrm{VOL}}$ or CTDI_vol_) [16,17].

Effective dose (*E*) is defined as a tissue-weighted sum of radiation-weighted tissue and organ doses of a reference model as a non-measurable derived quantity, introduced by the International Commission on Radiological Protection (ICRP), representing a measure of radiation detriment to reference models. Hence, its purpose is to be used for dose limits of stochastic effects in accordance with the radiation protection principles of occupationally exposed populations [18,19]. Although with certain limitations it can be used for radiation exposure of patients for the purpose of medical diagnosis. ICRP recommends that all procedures involving medical exposure should adhere to the principles of justification and optimization [20]. In CT, *E* is commonly estimated from DLP using Monte Carlo simulated conversion factors that are specific for an anatomical region of interest [21,22].

The estimated effective dose is the basis for radiation-induced cancer risk assessment. Clear evidence of radiation-induced cancer risk at doses above 100 mSv exists. The maximum dose generated by a single CT scan is far below 100 mSv. However, the cumulative radiation dose from multiple CT scans could easily reach this level. As a consequence, doses associated with computed tomography should be carefully considered [23]. We need to stress that we are always exposed to small doses of ionizing radiation from natural sources surrounding us. The amount of this so-called background radiation depends on many factors, such as altitude and ventilation for example. Under normal circumstances, exposure to ionizing radiation from natural or background sources does not vary a lot. Exposure increase is mostly due to CT scanning and nuclear imaging. Doses discussed in this paper are doses mostly delivered by radiation coming from the CT device to which patients were exposed. Delivered doses during a single conventional CT scan can be comparable to or in some cases higher than an annual effective dose from natural background radiation of about 3 mSv [24,25]. To analyse possible harmful influence of radiation on biological tissue in detail, various factors must be taken into account, including the age of the study population, type of rays, exposure characteristics and body parts exposed to radiation [25]. The previously introduced effective dose is only valid for comparing doses from different hospitals or countries, and it cannot be used for the detailed assessment of individual risk. So, several studies recommend replacing the effective dose with the risk of exposure-induced cancer death (REID) values which are based on age and gender [26]. The REID is defined as the probability that an individual will die from cancer associated with the exposure, and it can also be compared to other potential health risks in everyday life. As the most frequent cancers induced by different ionizing radiation, the scientific literature lists leukemia, thyroid cancer, bladder cancer, breast cancer, lung cancer, etc.

The purpose of this study was to assess the risk of exposure-induced death (REID) values in patients undergoing COVID-19-related chest computed tomography, as well as to evaluate the associated dose descriptors.

## 2. Materials and Methods

The study evaluated the dose data of 1801 CT chest procedures performed on patients who were admitted to the COVID-19 center “Podhrastovi” of the Clinical Centre of Sarajevo University in a 14-month period from April of 2020 to June of 2021. The main criteria for hospitalization were severe clinical deterioration, lung X-ray showing disease progression and low oxygen saturation with an SpO_2_ lower than 89%. As the data was collected by a software program (OpenREM), the patients remained anonymous, thus limiting our access to additional information, e.g., pregnancy, immunodeficiency, comorbidities, number of CT scans, disease outcome, etc. Because of that, corresponding approval from local ethical committee was not required. Patients were scanned on Toshiba Aquilion Lightning 16-row/32-slice CT scanner (Toshiba Corporation, Minato, Tokyo, Japan), using a clinically adjusted COVID-19 protocol.

Dose descriptors and accompanying procedure information were collected by the OpenREM dose monitoring system (The Royal Marsden NHS Foundation Trust, London, United Kingdom) which interprets radiation-structured dose reports (RDSR) stored on the picture archiving and communication system (PACS).

RDSR files contain detailed information on CT examination, including type of examination, date and time of the procedure, patient age and sex, exposure time, scan length, slice thickness, collimation width, pitch, tube potential (kV), maximum and mean tube current (mA) and rotation time, as well as the values of dose indices, namely air kerma length product (DLP) and pitch-corrected volume CT air kerma index (CTDI_vol_). Patients with incomplete data were excluded from the study.

Patient organ doses and effective doses were estimated using a CTVoxDos software package based on Monte Carlo simulations [27]. The obtained values were used as input for the PCXMC program (STUK, Helsinki, Finland) to calculate REID [28]. The risk of radiation-exposure-induced cancer in PCXMC was performed in accordance with the BEIR VII Committee, which represents an age- and sex-dependent model. Risk models are available for thyroid, liver, breast, ovaries, uterus, prostate, urinary bladder, lung, stomach, colon cancers, leukemia and for all other solid cancers combined [29].

The data was analysed using the IBM’s Statistical Package for Social Sciences (SPSS) version 26.0 (International Business Machines Corporation, Armonk, NY, USA). The significance level used in statistical calculations was set to α=0.05. The normality of the distribution was tested using the Kolmogorov–Smirnov test. In general, the dose data is not normally distributed. Hence, the nonparametric Mann–Whitney *U* test was used to assess the differences between data distributions.

## 3. Results

**Figure 1:**
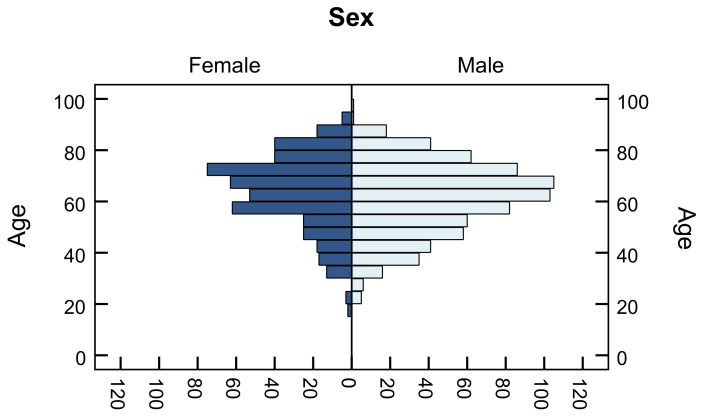
Age distribution relative to sex for patients who underwent chest CT scan with customized COVID-19 protocol.

Data were collected for 1801 but analysed for a total of 1180 adult patients. The reason for this is that for a certain number of patients there were no data on age and sex, so these data were not subject of statistical analysis. Age distribution relative to sex for COVID-19 patients who underwent chest CT scan with a special customized COVID-19 protocol is presented in Figure 1.

**Figure 2:**
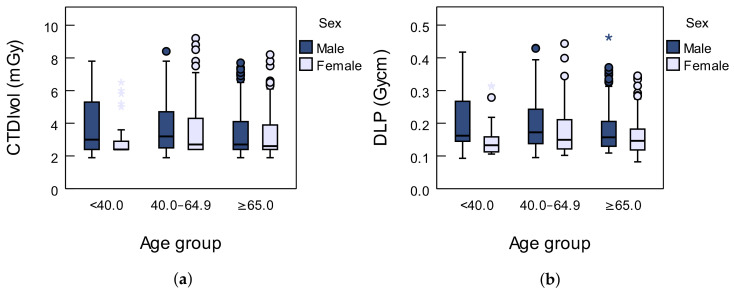
The box plot represents the distribution of dose descriptors for patients who underwent chest CT scan with special customized COVID-19 protocol: (**a**) CTDI_vol_; and (**b**) DLP. Outliers and extreme values are represented with circles and asterisks, respectively.

Among the patients, 61% were men, and 39% were women. Patients were divided into three age groups: younger than 40 years, between 40.0 and 64.9 years and older than 65 years. Values of dose descriptors were analyzed based on age and sex and presented in Table 1 and Figure 2a,b. Based on the results obtained with the adapted protocol, the corresponding data will be compared with the data obtained by using standard, low-dose (LDCT) and ultra-low-dose (ULDCT) protocols in the Section 4.

The median of CTDI_vol_ for male and female patients younger than 40 is 3.1 mGy and 2.4 mGy, respectively. For male and female patients between 40 and 64.9 years old, the median of CTDI_vol_ is 3.2 mGy and 2.7 mGy, respectively. The median of CTDI_vol_ for male and female patients older than 65 is 2.7 mGy and 2.6 mGy, respectively. In total, the median and interquartile range (ΔQ) of CTDI_vol_ for male and female patients are 3.0 mGy (ΔQ=1.9 mGy) and 2.6 mGy (ΔQ=1.5 mGy), respectively, for all age groups. The dose distribution is not normal (KolmogorovSmirnov test, p<0.001), and the difference between the two genders is significant (Mann–Whitney *U* test, p<0.001).

The median of DLP for male and female patients younger than 40 is 165 mGy cm and 136 mGy cm, respectively. For male and female patients between 40 and 64.9 years old, the median of DLP is 172 mGy cm and 149 mGy cm, respectively. The median of DLP for male and female patients older than 65 is 157 mGy cm and 146 mGy cm, respectively. In total, the median and interquartile range (ΔQ) of DLP for male and female patients were 164 mGy cm (ΔQ=92.1 mGy cm) and 147 mGy cm (ΔQ=71 mGy cm), respectively, for all age groups. Just like in the case of CTDI_vol_, the dose distribution is not normal (Kolmogorov–Smirnov test, p<0.001), and the difference between the two genders is significant (Mann–Whitney *U* test, p<0.001).

Dose descriptors are greater for male than for female patients for all age groups. The median and interquartile range values for CTDI_vol_ and DLP for both sexes and all age groups may be found in Table 1.

**Figure 3:**
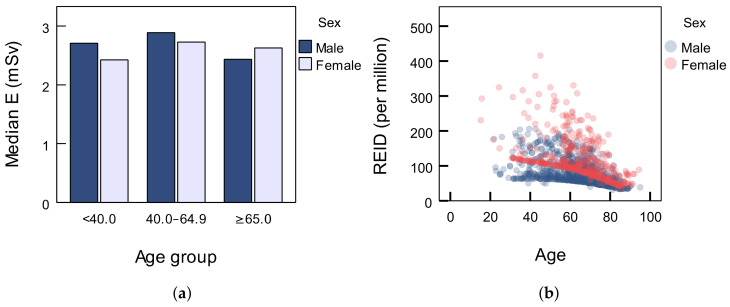
(**a**) Median effective dose relative to patients’ sex; (**b**) total radiation-exposure-induced death (REID) value for each patient versus age and relative to sex.

The median values of the effective dose *E* (mSv) for male and female patients are 2.7 mSv and 2.4 mSv for patients under the age of 40, 2.9 mSv and 2.7 mSv for patients between the age of 40 and 64.9 and 2.4 mSv and 2.6 mSv for patients aged 65 or older (see Table 2 and Figure 3a). The median value for the total number of patients is 2.6 mSv. Interquartile range values, ΔQ, for effective dose, *E*, for both sexes and all age groups may be found in Table 2. The distribution of effective doses is not normal (Kolmogorov–Smirnov test, p<0.001) and the difference between the two genders is significant (Mann–Whitney *U* test, p<0.001).

Data which are used in PCXMC are taken from ICRP 103 for the Euro-American population. The total REID values for each patient versus age and relative to sex are shown in Figure 3. The REID value decreases as age increases for both sexes (Pearson correlation test, r=−0.414,p<0.001). Unlike dose descriptors and effective dose, total REID is higher for female patients (Mann–Whitney *U* test, p<0.001). Additionally, the REID value difference between the sexes decreases as age increases. The greatest contributors to the total REID were breasts (younger females) and lungs, which can be seen in Table 3.

## 4. Discussion

In previous sections of the paper, we introduced and considered different protocols used for chest imaging [9,14]. The protocol which is used in 55% of COVID-19 CT imaging procedures is the routine chest CT protocol, followed by 43% with a low-dose protocol and 3% with a high-dose protocol, according to the IAEA [9]. Although a single CT scan does not pose a significant risk to the patient’s health, repeated procedures increase the risk of biological damage. Exposure to ionizing radiation should be carefully monitored, especially in children and young people because of long life expectancy and body development [30]. In their study, Sakane et al. stated that the standard protocol for chest CT with a standard dose of 5 mSv is associated with double-stranded DNA molecule bursting and chromosome aberrations [31]. Cristofaro et al. compared radiation doses for chest CT in COVID-19 patients and in a group of patients with pulmonary infectious diseases at the same time the year before. Analysis of these data showed an increase in the total mean dose in COVID-19 patients [32].

In this study, radiation dose descriptors in chest CT scans, CTDI_vol_ and DLP, as well as the effective dose *E* values for three age groups relative to sex, were presented. The difference in the value of dose descriptors by sex is significant according to the non-parametric Mann–Whitney *U* test (p<0.001), as it was stated earlier. In general, male patients are taller, so higher DLP is expected, and since automatic exposure control (AEC) is used, women receive lower doses [33]. Imaging methods without contrast media allow for faster procedures and reduced exposure of employees to the coronavirus. There are no specific (target) CT doses for COVID-19 patients, but the evaluation takes an upper limit of 3 mGy for CTDI_vol_, as recommended for low-dose CT screening of lung cancer patients [9]. The values of the total median for all age groups of CTDI_vol_ listed in Table 1 coincide with the stated limit for men, while for women it is lower than the stated value.

In the IAEA study on protocol and dose variations for 28 countries, the median values for CTDI_vol_ and DLP are higher than the same dose descriptors in this study, which is to be expected as more hospitals are included in the study. CTDI_vol_ and DLP median values (interquartiles range) for European countries were 8 (7) mGy and 321 (292) mGy cm, respectively. There were a total of 54 clinical hospital centers where CTDI_vol_ depended on the type of CT device, the number of rows of detectors, the year of installation of the device and reconstruction techniques. Multiphase CT examinations were performed in about 20% of clinics, so DLP had a higher value than in the case of single-phase examinations in 80% of clinics [10]. Steuwe et al. reported the range of values is 1.4–6.7 mGy for CTDI_vol_ and 48.5–201.7 mGy cm for DLP, with mean values of 2.8 mGy and 89.3 mGy cm, respectively. The purpose of the study was to evaluate protocol settings, radiation exposure, image quality and diagnostic efficacy of the low-dose CT protocol at a university clinic [34]. The time span for data collection was one month, so the number of CT scans for which doses were collected is significantly lower (105 scans) compared to the number of scans in our study. All tests were performed on the same CT device for a similar imaging protocol (100 kVp, 60 mAs, step 0.6) as in our paper. The total median value for CTDI_vol_ of our work is relatively close to the mean value they obtained, while the median value for DLP is much higher than the mean value given in Steuwe’s paper. On the other hand, Pan et al. reported significantly higher values for CTDI_vol_, mean value of 8.4 mGy and a range of 5.2–12.6 mGy for 21 patients, which is also expected given that the patients in that study were scanned an average of four times [35]. A Brazilian multicenter study reported CTDIvol and DLP median values (interquartiles range) of 10 (4) mGy and 367 (166) mGy cm and 9 (4) mGy and 298 (140) mGy cm for male and female patients, respectively. Since a routine, non-contrast chest CT protocol (standard protocol) was used in all healthcare centers, and since that included different types of scanners, it is understandable that dose descriptors were higher in comparison to ours [36]. A study focusing on 15 pregnant women who had pneumonia as a result of COVID-19 disease reported values for CTDI_vol_, mean values of 4.1 mGy and a range of 2.3–5.8 mGy [37]. The tests were performed on a low-dose CT device. However, the reported range value for CTDI_vol_ of this study is higher than our values for both sexes even though it was performed on pregnant women. Zhou et al. collected dose data from 92 hospitals for 550 patients. The median value for DLP was 325.2 mGy cm with a range of 6.79–1098 mGy cm, and the median value for CTDI_vol_ was 9.68 mGy, with a range of 0.62–33.80 mGy for scanning protocols in Chonqing, China [38]. These data are significantly higher than those in our study, but it is important to note that these were doses collected from 92 different hospital centers with different scan parameters and different CT devices. The authors state that protocol and dose assessment was performed at the individual level specifically for each patient and that for less than 1.13% of tests, there was a CTDI_vol_ value of 3 mGy. Low-dose CT (LDCT) and ultra-low-dose CT (ULDCT) have been discussed in some studies related to management of COVID-19 disease [39,40,41,42].

One study compared the reliability of lung LDCT (80 kV, 40mA) compared to the standard protocol (120 kV, 300mA). Based on these parameters, median values for DLP of 189.98 mGy cm and 15.59 mGy cm for standard and LDCT protocols were obtained [39]. An LDCT protocol with lower kV values and mA reduced the doses delivered to patients. The study points out that LDCT protocols should not be used in the initial diagnosis of COVID-19 when greater sensitivity is preferred, as the occurrence of consolidation is difficult to detect. The stated dose values for LDCT are much lower than the doses obtained in our study. One of the first studies, reported by Agostini et al., assessed higher pitch values for a dual-source CT device in COVID-19 patients. Although lung images show higher noise compared to the high-dose protocol, the impact on the detection and characterization of anatomical and pathological features and signal-to-noise ratio and contrast-to-noise ratio of CT images in lung diseases were not significant [42]. Schulze-Hagen et al. state that LDCT tests should be used in the diagnosis of COVID-19 because they provide sensitivities comparable to RT-PCR tests, and thus correct false-negative results, as well as in the diagnosis of patients with nonspecific clinical symptoms. The authors believe that in case of future pandemic waves and lack of RT-PCR tests, LDCT should be used to identify symptomatic patients infected with the SARS-CoV-2 virus [40]. Tawk et al. found that standard chest CT as well as low-dose CT are very sensitive tools for COVID-19 infection detection. However, it is showed that the specificity was very poor for the LDCT, which could be explained by the presence of a high false-negative rate of CT in the early stages of the disease. Nevertheless, the authors conclude that using the LDCT protocol achieved a 90% reduction in the estimated dose without losing diagnostic information on images [43]. The median values for CTDI_vol_ and DLP in our study for all age groups and both sexes are much lower than those of most of the above-reported standard CT lung examinations but much higher than in the case of the reported LDCT values. The International Commission on Radiological Protection (ICRP) introduced the concept of diagnostic reference levels (DRL) in 1996 in report no. 73, and in 2001 practical guidelines were given, while in 2017, report no. 135 further clarified the terminology and sizes used to establish the DRLs. DRLs are typically placed at the 75th percentile of the dose distribution from a broad, national study using a set protocol [44].The European Commission report states DRL values for 36 European countries, and DRL values for chest CT scans are 400 mGy cm for DLP (range 270–700 mGy cm) and 10 mGy values for CTDI_vol_ (range 10–30 mGy) [45]. The American College of Radiology (ACR) has issued a DRL value of 12 mGy for chest CT scans without contrast agent for CTDI_vol_. In addition, they suggest that for LDCT, this value must be less than 3 mGy, which is close to our results. Of the total number of examinations of our study, the median values for DLP and CTDI_vol_ are below the DRLs reported in the European Commission report.

The calculated median value of an effective dose *E* (2.6 mSv) is below the value reported by Zhou et al. 4.55 mSv, but above the values reported for low-dose computed tomography examinations such as 0.28 mSv by Agostini et al., 1.3 mSv by Stuewe et al. and 0.56 mSv by Dangis et al. [34,38,42,46]. It is important to point out that in these studies the effective dose was calculated using the conversion factor and the DLP value, while we calculated the effective dose in the CTVoxDos program.

One of the shortcomings of our study, as mentioned earlier, is that the data were taken from only one healthcare unit (a single 16-slice CT scanner), which limits the comparison and thus the generalization. In addition to the above, apart from data on doses, gender and age, we did not have any data on disease severity or clinical features, along with no information about follow-up scans or multiphase axaminations, and no analysis of the obtained CT images was performed. In addition, there was no data on patient mass, which prevented the use of BMI-based guidelines in the process of optimization and more sophisticated statistical analysis. The reasons are data privacy and electronic data collection. On the other hand, it is crucial to have access to electronic data collection systems (e.g., OpenREM), which contributes to greater accuracy of dose assessment and enables faster data collection for a large number of patients.

To date, there is almost no (or little) information about risk-associated quantities for patients undergoing COVID-19-related chest CTs with adjusted protocols. Damage to human cells due to exposure to ionizing radiation can cause cancer in patients undergoing CT scans, where age and dose level affect the risk of cancer. Adults show a higher COVID-19 infectivity rate than children and younger individuals [47,48]. Easy acquisition and availability of CT may result in avoidable exposure of patients, especially if the imaging procedure is not optimized. In addition, multiphase CT procedures are not necessary for most clinical indications, especially for routine chest CT [49]. The cancer risk caused by moderate to high doses of ionizing radiation is well known; however, the risks from low-dose sources, most of which include CT, are still controversial. Although most epidemiological studies have approved the linear no-threshold (LNT) model for risk prediction, an increasing number of studies emphasized inherent uncertainty in the LNT model [50,51,52,53]. Although the risk in an individual patient is relatively small and the background risk of cancer in the population relatively high, it is very challenging to conduct an epidemiological study with a sufficient number of patients that would enable precise risk estimates [50]. An LNT model should be used combined with general optimization strategies in order to balance the uncertain risk of cancer induction versus diagnostic benefits [53]. In addition to dose descriptors and DRLs, it is necessary to emphasize the importance of REID values estimation for referring medical doctors and radiologists to be able to evaluate the associated risk to patients. PCXMC software has been used for the estimation of REID values in various studies [26,54,55,56], but correlation was difficult since there was no research addressing values for this type of procedure. Younger females are at higher risk due to greater radiosensitivity and position of certain organs, especially breasts as displayed in Table 3 and Figure 3b. The limitation of this approach to risk evaluation is that there is no experimental research which proves validation of the linear no-threshold model, mentioned as well in similar risk assessment studies [26,54]. In accordance with all the above statements, we did not compare our results with the abovementioned studies, especially because it is not the same diagnostic procedure.

## 5. Conclusions

In our study, we found that median values of defined dose descriptors (CTDI_vol_ and DLP) are greater for male than for female patients for all age groups. Based on the abovementioned values of dose descriptors, patient organ doses and effective doses were estimated. Using such obtained data as input, assessment of risk of exposure-induced death (REID) was obtained. In accordance with the applied model, younger females are at higher risk due to greater radiosensitivity and position of certain organs.

There are a few limitations of the presented study. One of them is that all data are taken from one regional center. We did not have any data on disease severity or clinical features, along with no information about follow-up scans or multiphase examinations, and no analysis of the obtained CT images was performed. The absence of information related to the anatomical features of patients (mass, height, etc.) prevented a wider statistical analysis. Furthermore, there are limitations when determining the REID values as well, due to the inherent uncertainty of the LNT model.

For further study, we consider expansion of the conducted research to other centers in the region, as well as the possibility to introduce data related to the follow-up scans, patient anatomy (i.e., body diameter) and image quality (i.e., image contrast and noise) on the existing data set.

We are already in the third year since the start of the COVID-19 pandemic was declared. Although the pandemic seems to be fading, scientists should keep a close eye on the situation. It is important to further evaluate the risks and benefits of chest CT scans in the context of COVID-19.

## Figures and Tables

**Table 1:** Median value $\tilde{x}$ and interquartile range ΔQ of $C_{\mathrm{VOL}}$ and *P*_KL,CT_ values for male and female patients relative to gender for three age groups.

		CTDI_vol_ (mGy)				DLP (mGy cm)			
		**Male**		**Female**		**Male**		**Female**	
		$\tilde{x}$	ΔQ	$\tilde{x}$	ΔQ	$\tilde{x}$	ΔQ	$\tilde{x}$	ΔQ
Age group	<40.0	3.1	3.0	2.4	0.75	165	123	136	46
	40.0–64.9	3.2	2.2	2.7	1.9	172	105	149	90
	≥65.0	2.7	1.7	2.6	1.5	157	75.8	146	64
	Total	3.0	1.9	2.6	1.5	164	92.1	147	71

**Table 2:** Median $\tilde{x}$ and interquartile range ΔQ of effective dose *E* for male and female patients relative to gender for 3 age groups.

		*E* (mSv)					
		**Male**		**Female**		**Total**	
		$\tilde{x}$	ΔQ	$\tilde{x}$	ΔQ	$\tilde{x}$	ΔQ
Age group	<40.0	2.7	2.6	2.4	0.6	2.6	1.8
	40.0–64.9	2.9	2.0	2.7	1.9	2.8	1.9
	≥65.0	2.4	1.5	2.6	1.5	2.5	1.4
	Total	2.7	1.7	2.6	1.5	2.6	1.5

**Table 3:** Radiation-exposure-induced death (REID) values relative to age groups and sexes for specific organs.

Mean REID Values (per Million)										
**Age**	**Sex**	**Leukemia**	**Breast**	**Colon**	**Liver**	**Lung**	**Ovary**	**Stomach**	**Bladder**	**Total**
<40.0	M	12.8	-	27.1	9.1	42.3	-	13.4	0.4	105.1
	F	9.4	29.3	3.9	6.4	92.5	0.1	19.1	0.1	160.9
40.0–64.9	M	13.8	-	23.2	7.6	38.1	-	11.5	0.4	94.5
	F	11.7	9.5	3.8	6.2	92.9	0.1	18.6	0.1	142.9
≥65.0	M	12.4	-	15.3	4.4	27.0	-	7.3	0.4	66.7
	F	10.1	2.3	2.5	3.7	59.6	0.0	12.0	0.1	89.6
Total	M	13.1	-	20.1	6.3	33.6	-	9.8	0.4	83.3
	F	10.7	7.2	3.1	4.9	75.4	0.1	15.2	0.1	116.3
TOTAL		12.1	7.2	13.5	5.8	49.9	0.1	11.9	0.3	96.1

## Data Availability

Not applicable.
